# Leveraging large language models for academic conference organization

**DOI:** 10.1038/s41746-025-01492-7

**Published:** 2025-02-14

**Authors:** Yuan Luo, Yikuan Li, Omolola Ogunyemi, Eileen Koski, Blanca E. Himes

**Affiliations:** 1 Department of Preventive Medicine, Feinberg School of Medicine, Northwestern University, Chicago, IL USA; 2 Northwestern University Clinical and Translational Sciences Institute, Feinberg School of Medicine, Northwestern University, Chicago, IL USA; 3 Center for Collaborative AI in Healthcare, Institute for AI in Medicine, Feinberg School of Medicine, Northwestern University, Chicago, IL USA; 4 Center for Biomedical Informatics, Charles R. Drew University of Medicine and Science, Los Angeles, CA USA; 5 IBM T.J. Watson Research Center, Yorktown Heights, NY USA; 6 Department of Biostatistics, Epidemiology and Informatics, University of Pennsylvania, Philadelphia, PA USA

**Keywords:** Conferences and meetings, Medical research

## Abstract

We piloted using Large Language Models (LLMs) for organizing AMIA 2024 Informatics Summit. LLMs were prompt engineered to develop algorithms for reviewer assignments, group presentations into sessions, suggest session titles, and provide one-sentence summaries for presentations. These tools substantially reduced planning time while enhancing the coherence and efficiency of conference organization. Our experience shows the potential of generative AI and LLMs to complement human expertise in academic conference planning.

## Introduction

Organizing an academic conference poses numerous logistical and intellectual challenges, especially as conferences grow in size and scope. Core tasks such as matching submissions with qualified reviewers, organizing sessions around coherent themes, and summarizing presentations require significant time and meticulous coordination. As the volume and diversity of submissions increase, these tasks become even more labor-intensive, and the risk of inconsistencies or errors grows. These demands highlight the need for innovative approaches that can streamline conference organization while maintaining high standards of coherence and accessibility.

**Fig. 1:**
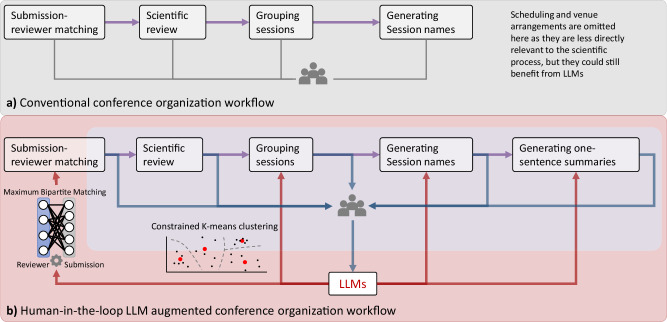
Innovative approaches to conference organization scientific process. **a** Conventional workflow. **b** LLM-Augmented workflow. LLMs can be used to assist with submission-reviewer matching, grouping presentations into sessions, generating session names, and generating one-sentence summaries for presentations. To uphold the integrity of the scientific review process, note that LLMs are excluded from directly impacting reviewers’ ratings on submissions.

In response to these challenges, the AMIA 2024 Informatics Summit Scientific Program Committee piloted a new approach using generative artificial intelligence (genAI) and Large Language Models (LLMs), while utilizing only publicly available information about submissions. For this conference, submissions include podium abstracts and papers that are programmed as presentations and abstracts that are programmed as posters. Our initiative explored how prompt-engineered LLMs could assist in various facets of conference organization to enhance both efficiency and participant experience. Specifically, we leveraged LLMs to facilitate algorithmic enhancement of submission-reviewer matching, thematic grouping of presentations, and generating one-sentence summaries for each presentation to aid attendee navigation (Fig. 1).

This early attempt to integrate LLMs into conference planning aimed at both reducing organizational burdens and improving the thematic coherence and clarity of information provided to attendees. Importantly, we ensured that LLMs did not generate content that could influence reviewers’ evaluations, preserving the integrity of the review process. Our approach reflects an initial step toward harnessing AI-driven tools to complement human expertise in conference management, providing insights that may inform future systematic studies on the role of AI in academic conference settings.

## Using LLMs to facilitate academic conference programming

The integration of LLMs into the AMIA 2024 Informatics Summit began with leveraging an LLM to develop an advanced algorithm for submission-reviewer matching. This algorithm included email domain conflict checks and keyword matches between submissions and reviewers, where submission authors and reviewers were asked to pick from the same set of keywords in the AMIA submission portal to ensure accurate matching. This approach significantly reduced time and effort while maintaining high accuracy in reviewer assignments. Another key innovation was the thematic grouping of presentations and posters. By analyzing titles and abstracts with LLMs, we efficiently identified thematic groupings, ensuring sessions were relevant and facilitated focused discussions. This method replaced traditional manual curation, which can be time-consuming and less precise in capturing nuanced connections between presentations. Additionally, LLMs generated concise one-sentence summaries for each presentation, providing attendees with a quick understanding of key points. These summaries were included in the online program, enhancing the navigability of the conference and allowing participants to make informed and timely decisions about which sessions to attend. This innovation was particularly valuable for AMIA’s multidisciplinary offerings, making the conference more accessible and engaging.

This study does not constitute human subjects research, as no identifiable or non-public information from submissions and presentations was used by LLMs. AMIA leadership and staff were involved throughout the process, and we had their permission to use the data for this purpose.

1. When using the LLM to write initial Python code for paper-reviewer matching, the LLM was only instructed to generate generic code and did not access any specific paper or reviewer data. It neither viewed nor directly matched any paper with a reviewer.
2. For grouping presentations into sessions, the LLMs accessed only the titles, abstracts, and keywords of accepted presentations, which are publicly available information and contain no PHI or PII.
3. The one-sentence summaries were generated using only the title and abstract of each presentation, both of which are publicly available data.

## Algorithmic enhancement of submission-reviewer matching with LLMs

Assigning submissions to reviewers has always been challenging due to the need to match topics accurately, manage conflicts of interest, and balance workloads. Automated systems like the Toronto Paper Matching System (TPMS)^1^ are often used but can fall short in meeting the specific needs of each conference, such as prioritizing diversity and detailed expertise alignment. Furthermore, the “one-size-fits-all” approach may not handle unique fields or emerging research areas with sparse literature. Thus, exploring automated ways to develop custom systems can offer more control over matching criteria and the flexibility to adapt to the evolving needs of the academic community.

We adopted the chain-of-thought and human-in-the-loop approaches and began with the first prompt stating the number of reviewers (*m*) and papers (*n*), each with a list of keywords. This prompt asked ChatGPT for an algorithm to match papers to reviewers based on keyword overlap, ensuring no reviewer received more than *r* papers. ChatGPT^2^ suggested the Hungarian Algorithm or the Maximum Bipartite Matching Algorithm with additional constraints, and recommended the SciPy^3^ and NetworkX^4^ packages. We verified that the suggested Maximum Bipartite Matching Algorithm ensured maximum keywords overlap between matched reviewers and submissions^5^. This algorithm treated papers and reviewers as two distinct sets of nodes (hence bipartite) in a graph, with edges representing possible assignments weighted by expertise keywords overlap. The algorithm paired each paper with a reviewer in a way that maximized the total expertise overlap (edge weights) of assignments, with no reviewer assigned more papers than the *r* allowed. We described the data structure in our second prompt for ChatGPT to write Python code to construct a reviewer-submission bipartite graph (reviewers should be duplicated *r* times) using the NetworkX package. We specified that a reviewer-submission edge cannot exist if the reviewer’s email domain overlapped those contained in the submission. In our third prompt, we specified that the code should count shared keywords between a reviewer and a submission for edge weights and use the Maximum Bipartite Matching Algorithm for reviewer-submission matching. We verified the Python code generated by ChatGPT line-by-line and found no errors, then ran it and post-processed the results for downstream use.

## Grouping presentations and posters into thematic sessions

The structure of academic conferences usually revolves around a central theme with sessions consisting of presentations grouped according to topic similarity. Traditionally, organizing committees manually curate these themes and group presentations. However, the scale and complexity of modern academic gatherings demands automated solutions. Recent developments in recommender systems, such as those reviewed by Beel et al.^6^ and techniques like topic modeling, used for grouping posters by the Society of Neuroscience Conference^7^, have improved organizing the scientific literature. These methods, however, struggle with fine resolution sub-themes and emerging topics that are not well-represented in existing data. LLMs analyze and categorize vast textual data more effectively than earlier methods like topic modeling^2,8,9^, enabling more nuanced session organization by capturing the essence and thematic connections of academic presentations.

We concatenated the titles and abstracts of submissions for a comprehensive representation and processed the combined texts using the embedding model text-embedding-ada-002 available through OpenAI’s API to generate embeddings. These embeddings numerically represented the semantic content, capturing the nuanced information of each submission. With the embeddings prepared, we applied a constrained K-means clustering algorithm^10^ to ensure each session contained exactly six submissions, adhering to size constraints on the number of presentations each session could accommodate. This method grouped thematically homogeneous papers while meeting logistical needs. The conference Chair (YLuo) and Vice Chairs (BEH, EK, and OO) reviewed these groupings and made any adjustments that were deemed necessary. We then used Python scripts to prompt ChatGPT to create session names by instructing it to create “a concise, catchy, and reflective title” using the concatenated titles and abstracts of the six presentations for each session. These names were reviewed and revised by the Chair and Vice Chairs, who ensured they accurately reflected the content of the submissions while being engaging and informative. These steps combined the efficiency of LLMs with human expertise in a human-in-the-loop system.

## Informative one-sentence summaries for presentations

To help attendees identify sessions of interest and navigate the conference program, we used LLMs to generate concise one-sentence summaries for each presentation, based on provided abstracts. This approach distilled complex research findings into single, digestible sentences, allowing attendees to quickly grasp key points. These summaries significantly enhanced the educational value and navigability of the conference by enabling participants to efficiently manage the wealth of information presented. Presenting academic research succinctly is challenging, especially in settings with diverse topics and time constraints. Traditional abstracts are informative but often require considerable time to understand fully. In contrast, LLM-generated one-sentence summaries capture the essence of each presentation succinctly. This saves attendees’ time and aids in making informed decisions about which sessions to attend based on their interests and research needs.

We obtained summaries by first joining the titles and abstracts of each submission into a single string, while clearly delineating their boundaries with new lines and prepending them with “Title:” and “Abstract:”, respectively. This consolidated text was then processed using ChatGPT (model gpt-3.5-turbo-1106) with the prompt to produce a concise one-sentence summary for each submission. Summaries were split and reviewed by the authors.

## Discussion

The use of LLMs to help organize the AMIA 2024 Informatics Summit highlights their potential to support and enhance human decision-making in academic environments. By automating routine and complex data processing tasks, LLMs freed organizers and attendees to engage more deeply with the substantive content of the conference. This improved logistical efficiency and academic discourse during the conference by ensuring there was thematic coherence and facilitating conference navigation.

We demonstrated that it is possible to use LLMs to establish a submission-reviewer matching system with customized requirements and addressed the following limitations with a human-in-the-loop approach. First, using email domains alone may not fully capture potential conflicts, particularly when reviewers or authors use personal email addresses or when co-author affiliations are not directly accessible. To address this limitation, we implemented a multistep, human-in-the-loop approach to strengthen COI safeguards. First, after applying the automated email domain-based exclusion, we included a manual review step where organizers assessed the matches to identify any potential conflicts missed by the automated process. Additionally, reviewers were asked to identify and disclose any conflicts for papers assigned to them, which was the traditional way to identify conflicts in prior AMIA conferences. Our layered human-in-the-loop approach provides a balanced strategy that combines automated efficiency with human judgment to better ensure COI compliance. Second, reviewer expertise in our matching process was based on self-reported keywords. For more than a decade, AMIA has maintained a pool of reviewers and keywords that is continually updated by retaining those reviewers who demonstrate productivity and reliability, as evidenced by their responsiveness and quality in reviewing assignments aligned with their self-reported keywords in previous years. While this approach has proven effective, we acknowledge its limitations, as the accuracy of reviewer assignments inherently depends on the precision and relevance of the keywords reported. To address this, we incorporated a human-in-the-loop process where conference organizers reviewed the automated matches, making adjustments as needed to ensure optimal reviewer assignments. This combination of self-reported expertise, data-informed matching, and human oversight represents a balanced approach and is consistent with best practices in the field.

The thematic grouping of presentations, powered by LLMs, enabled the creation of intellectually stimulating and contextually relevant sessions. By effectively capturing the essence and thematic connections of academic papers, LLMs facilitated the creation of sessions that resonated with the intended sub-themes of the conference. Moreover, voluntary feedback by multiple attendees during the conference indicated that the one-sentence summaries improved their experience. Specifically, attendees noted that these concise summaries were easy to read on mobile devices and provided quicker comprehension compared to reading full abstracts, helping them to more efficiently decide which sessions to attend. In the future, a systematic evaluation of the potential benefits of an automated one-sentence summarization approach in multidisciplinary conferences is needed to determine how to best assist participants in navigating concurrent sessions.

The level of human intervention required for LLMs varies across tasks. Grouping presentations and posters into thematic sessions demanded the most human oversight. For submission-reviewer matching, stepwise chain-of-thought prompting helped select the core algorithm, define the data structure, encode constraints, and implement the chosen algorithm using the appropriate packages. This resulted in effective matches without reviewer complaints. Generating one-sentence summaries required minimal human revision, as the organizers found that LLMs produced good summaries. Although logistical constraints prevented A/B testing, we can provide an estimated comparison based on our experience. Using the LLM-assisted approach, prompting ChatGPT to generate Python code for reviewer-submission matching, inspecting, and running it took around 30 minutes in total. In contrast, the traditional manual approach during the previous year’s AMIA Informatics Summit (slightly smaller in size) required over 20 h (YLuo was Vice Chair). For grouping presentations into sessions, writing and executing Python code using the OpenAI API took around 30 min, with an additional two hours for organizers to refine the grouping and session names. By comparison, manually creating session groupings for the previous year’s summit took approximately 2.5 days. Similarly, generating one-sentence summaries for presentations using the LLM-assisted approach required around 15 min to write and execute the Python code. Manually summarizing 150 presentations—assuming 10 min per summary—would take approximately 25 h. While splitting and reviewing the LLM-generated summaries added about two hours for the authors, this review process could be delegated to respective authors in future conferences, further distributing the time cost. These comparisons highlight substantial time savings with the LLM-assisted approach, showing potential as an efficient alternative to traditional methods.

For the task of grouping presentations and posters into thematic sessions, we assessed the consistency of clustering results from two LLMs using different inputs (titles, abstracts, and keywords). Due to logistical and timeline constraints inherent to conference organization, we were unable to exhaustively test every possible configuration and permutation for each LLM. Instead, we focused on a limited set of input configurations with practical relevance for GPT and LLaMa models. Table 1 shows that our GPT-based models were more consistent with varying inputs compared to LlaMa models, which showed low consistency. The highest consistency for GPT was between title-and-abstract and title-abstract-keyword inputs, but even this was modest, indicating sensitivity to input and the need for human oversight. Based on these comparisons—and following consensus among the Chair and Vice Chairs—we found that GPT-based results were generally preferable for our specific needs. We recognize, however, that this conclusion is limited by the scope of our comparison and is subject to the specific preferences and organizational context of this conference.

**Table 1:** Clustering consistencies among various configurations of different LLMs

		text-embedding-ada-002			AoE		
		Ti	TiAb	TiAbKw	Ti	TiAb	TiAbKw
text-embedding-ada-002	Ti	-	0.2465	0.1803	0.0558	0.0292	0.0659
	TiAb		-	0.3189	0.0235	0.0637	0.1300
	TiAbKw			-	0.0271	0.0558	0.1086
AoE	Ti				-	0.0062	0.0177
	TiAb					-	0.1393
	TiAbKw						-

We experimented with two LLM embedding models (text-embedding-ada-002 based on GPT architecture and angle-optimized text embeddings AoE^11^ based on LlaMa 2) with different combinations of title (Ti), abstract (Ab) and keywords (Kw) for presentations to be clustered. We calculated the adjusted mutual information (AMI) between the clustering results, score between 0 and 1 reflecting their agreement with each other (higher score indicates more agreement).

Organizers selected the title-and-abstract clustering by GPT after review. Adding keywords sometimes led to generic groupings, whereas excluding them kept submissions more topic-specific. For example, a study on transgender populations in pediatric psychiatry, tagged with the keyword Social Determinants of Health (SDOH), focused on EHR data characterization of subpopulations rather than SDOH itself. Further refinements were made to GPT’s clustering. For instance, multiple sessions on Natural Language Processing (NLP) needed reorganization into sub-themes like health language models and GPT-focused studies. The final sessions showed an adjusted mutual information (AMI) of 0.6041 with GPT’s suggestions, which was higher than the AMIs among all LLM-generated sessions, underscoring the importance that a good starting point provided by LLM could aid in human adjustments.

For many GPT-suggested session titles, we directly adopted the suggestions, such as “Empowering Clinical NLP with Large Language Models” and “SDoH: Insights, Interpretations, and Innovations.” However, some sessions required more human input to ensure the titles accurately reflected the diversity of their content. For example, we changed “Enhancing Interoperability and Clinical Decision Support with FHIR” to “Enhancing Interoperability in Healthcare” since not all presentations in that session focused on FHIR (Fast Healthcare Interoperability Resources) and clinical decision support. This ensured that session titles were inclusive and representative of their respective presentations. To assess the similarity between the lists of session names before and after human modification, we use GPT’s embedding models to generate vector representations of each session name in both lists. By comparing the cosine similarities between these vectors, we found the closest match for each session name in one list to a session name in the other list. The average of these similarities across all sessions had a numeric similarity score of 0.9334, demonstrating that minimal adjustments were deemed necessary.

In summary, this work represents an early effort to integrate LLMs into the conference organization process, providing valuable lessons for future academic conferences aiming to combine human expertise with the computational power of LLMs. Our approach is scalable and adaptable for leveraging AI in academic settings, ultimately fostering a more efficient and collaborative scholarly community. While LLMs have significantly advanced the automation of conference organization tasks like submission-reviewer matching and generating one-sentence summaries, human oversight remains essential, particularly in grouping presentations into thematic sessions. Complete automation of conference agenda creation with minimal human input calls for more iterative developments, continuous refinement and extensive testing as AI models become more sophisticated.

## Data Availability

Data for this study is available at https://github.com/luoyuanlab/LLM_Conference.

## Code availability

Code for this study is available at https://github.com/luoyuanlab/LLM_Conference.
